# P15 *Elizabethkingia anophelis* became the major *Elizabethkingia* species in bacteraemia patients in Northern Taiwan

**DOI:** 10.1093/jacamr/dlac133.019

**Published:** 2023-01-19

**Authors:** Fu-chieh Chang, Chang-Pan Liu, Chih-Chen Lin

**Affiliations:** Infection Control Center, Mackay Memorial Hospital, Taiwan; Mackay Junior College of Medicine, Nursing and Management, Taiwan; Infection Control Center, Mackay Memorial Hospital, Taiwan; Department of Infection, Mackay Memorial Hospital, Taiwan; Infection Control Center, Mackay Memorial Hospital, Taiwan; Department of Infection, Mackay Memorial Hospital, Taiwan

## Abstract

**Background:**

*Elizabethkingia* species are antibiotic-resistant microbes. It is a serious bacterial pathogen that can cause the patient to die. Thanks to the technology of sequencing, we can identify different strains of *Elizabethkingia* species. The *Elizabethkingia* genus currently comprises 6 species, *E. meningoseptica*, *E. miricola*, *E. anophelis*, *E. bruuniana*, *E. ursingii* and *E. occul*. In the previous study in Taiwan, *E. meningoseptica* was the major strain that caused infection from 2009 to 2016. But in another reference, we found that *E. anophelis* became the dominant strain in other countries. Due to this ascertainment, we did an experiment to check the true situation in Taiwan.

**Methods:**

Because of the limitation of the tool, in our hospital, we could spread different strains of Elizabethkingia Species by the traditionally identified methods in our bacteria room. That's why we collected the bacteria to our bacteria bank for research. In this study, we collected 65 *Elizabethkingia* species from bacteraemia patients. And the storage period from January 2018 to June 2020. Then we used the DNA extraction kit to collect the DNA and did the PCR to check whether the target gene was detected or not. Once *anoR* gene was detected, it would be *E. anophelis*, if the *mengF* gene was detected, it would be *E. meningoseptica*, or if the *ureGF* gene was detected, it would be *E. miricola*.

**Results:**

In this study, we found *E. anophelis* became the major species (37/65, 56.9%), and *E. meningoseptica* is the second most common species (28/65, 43.1%). We didn't find any *E. miricola*.

**Conclusions:**

The transmission route of *E. anophelis* remains unclear. The case-fatality rate of patients with *E. anophelis* infection is critically high, ranging from 24% to 60%. To avoid hospital associated infection with *E. anophelis*, environment cleaning plays an important role. In a recent study, UVC machines can reduce the infection rate, and may be another solution for environment cleaning besides bleach wipes.

